# Minimally invasive versus open pancreatoduodenectomy for pancreatic and peri-ampullary neoplasm (DIPLOMA-2): study protocol for an international multicenter patient-blinded randomized controlled trial

**DOI:** 10.1186/s13063-023-07657-7

**Published:** 2023-10-12

**Authors:** Nine de Graaf, Anouk M. L. H. Emmen, Marco Ramera, Bergthor Björnsson, Ugo Boggi, Caro L. Bruna, Olivier R. Busch, Freek Daams, Giovanni Ferrari, Sebastiaan Festen, Jony van Hilst, Mathieu D’Hondt, Benedetto Ielpo, Tobias Keck, Igor E. Khatkov, Bas Groot Koerkamp, Daan J. Lips, Misha D. P. Luyer, J. Sven D. Mieog, Luca Morelli, I. Quintus Molenaar, Hjalmar C. van Santvoort, Mirjam A. G. Sprangers, Clarissa Ferrari, Johannes Berkhof, Patrick Maisonneuve, Mohammad Abu Hilal, Marc G. Besselink, for the European Consortium on Minimally Invasive Pancreatic Surgery (E-MIPS)

**Affiliations:** 1 Department of General Surgery, Fondazione Poliambulanza Istituto Ospedaliero, Brescia, 25123 Italy; 2 Department of Surgery, Amsterdam UMC, location University of Amsterdam, Amsterdam, the Netherlands; 3 Cancer Center Amsterdam, Amsterdam, the Netherlands; 4 Department of Surgery, Linköping University Hospital, Linköping, Sweden; 5 Department of Surgery, Universitá Di Pisa, Pisa, Italy; 6 Department of Surgery, Niguarda Ca’Granda Hospital, Milan, Italy; 7 Department of Surgery, OLVG, Amsterdam, the Netherlands; 8 Department of Surgery, AZ Groeninge, Kortrijk, Belgium; 9 Department of Surgery, Hospital del Mar, Barcelona, Spain; 10 Department of Surgery, UKSH Campus Lübeck, Lübeck, Germany; 11 Department of Surgery, Moscow Clinical Scientific Center, Moscow, Russian Federation; 12 Department of Surgery, Erasmus MC, Rotterdam, the Netherlands; 13 Department of Surgery, Medisch Spectrum Twente, Enschede, the Netherlands; 14 Department of Surgery, Catharina Ziekenhuis, Eindhoven, the Netherlands; 15 Department of Surgery, Leiden University Medical Center, Leiden, the Netherlands; 16 General Surgery Unit, Department of Translational Research and New Technologies in Medicine and Surgery, University of Pisa, Pisa, Italy; 17 Department of Surgery, University Medical Center Utrecht, Utrecht, the Netherlands; 18 Department of Surgery, St. Antonius Hospital, Nieuwegein, the Netherlands; 19 Department of Medical Psychology, Amsterdam UMC, University of Amsterdam, Amsterdam, the Netherlands; 20 Department of Epidemiology and Data Science, Amsterdam UMC, VU University, Amsterdam, the Netherlands; 21 Division of Epidemiology and Biostatistics, IEO European Institute of Oncology IRCCS, Milan, Italy

**Keywords:** Minimally invasive, Laparoscopic, Robot-assisted, Pancreatoduodenectomy, Whipple, Pancreatic surgery, Pancreatic cancer, Peri-ampullary cancer, Pancreatic ductal adenocarcinoma

## Abstract

**Background:**

Minimally invasive pancreatoduodenectomy (MIPD) aims to reduce the negative impact of surgery as compared to open pancreatoduodenectomy (OPD) and is increasingly becoming part of clinical practice for selected patients worldwide. However, the safety of MIPD remains a topic of debate and the potential shorter time to functional recovery needs to be confirmed. To guide safe implementation of MIPD, large-scale international randomized trials comparing MIPD and OPD in experienced high-volume centers are needed. We hypothesize that MIPD is non-inferior in terms of overall complications, but superior regarding time to functional recovery, as compared to OPD.

**Methods/design:**

The DIPLOMA-2 trial is an international randomized controlled, patient-blinded, non-inferiority trial performed in 14 high-volume pancreatic centers in Europe with a minimum annual volume of 30 MIPD and 30 OPD. A total of 288 patients with an indication for elective pancreatoduodenectomy for pre-malignant and malignant disease, eligible for both open and minimally invasive approach, are randomly allocated for MIPD or OPD in a 2:1 ratio. Centers perform either laparoscopic or robot-assisted MIPD based on their surgical expertise. The primary outcome is the Comprehensive Complication Index (CCI®), measuring all complications graded according to the Clavien-Dindo classification up to 90 days after surgery. The sample size is calculated with the following assumptions: 2.5% one-sided significance level (α), 80% power (1-β), expected difference of the mean CCI® score of 0 points between MIPD and OPD, and a non-inferiority margin of 7.5 points. The main secondary outcome is time to functional recovery, which will be analyzed for superiority. Other secondary outcomes include post-operative 90-day Fitbit™ measured activity, operative outcomes (e.g., blood loss, operative time, conversion to open surgery, surgeon-reported outcomes), oncological findings in case of malignancy (e.g., R0-resection rate, time to adjuvant treatment, survival), postoperative outcomes (e.g., clinically relevant complications), healthcare resource utilization (length of stay, readmissions, intensive care stay), quality of life, and costs. Postoperative follow-up is up to 36 months.

**Discussion:**

The DIPLOMA-2 trial aims to establish the safety of MIPD as the new standard of care for this selected patient population undergoing pancreatoduodenectomy in high-volume centers, ultimately aiming for superior patient recovery.

**Trial registration:**

ISRCTN27483786. Registered on August 2, 2023

## Background

**Fig. 1:**
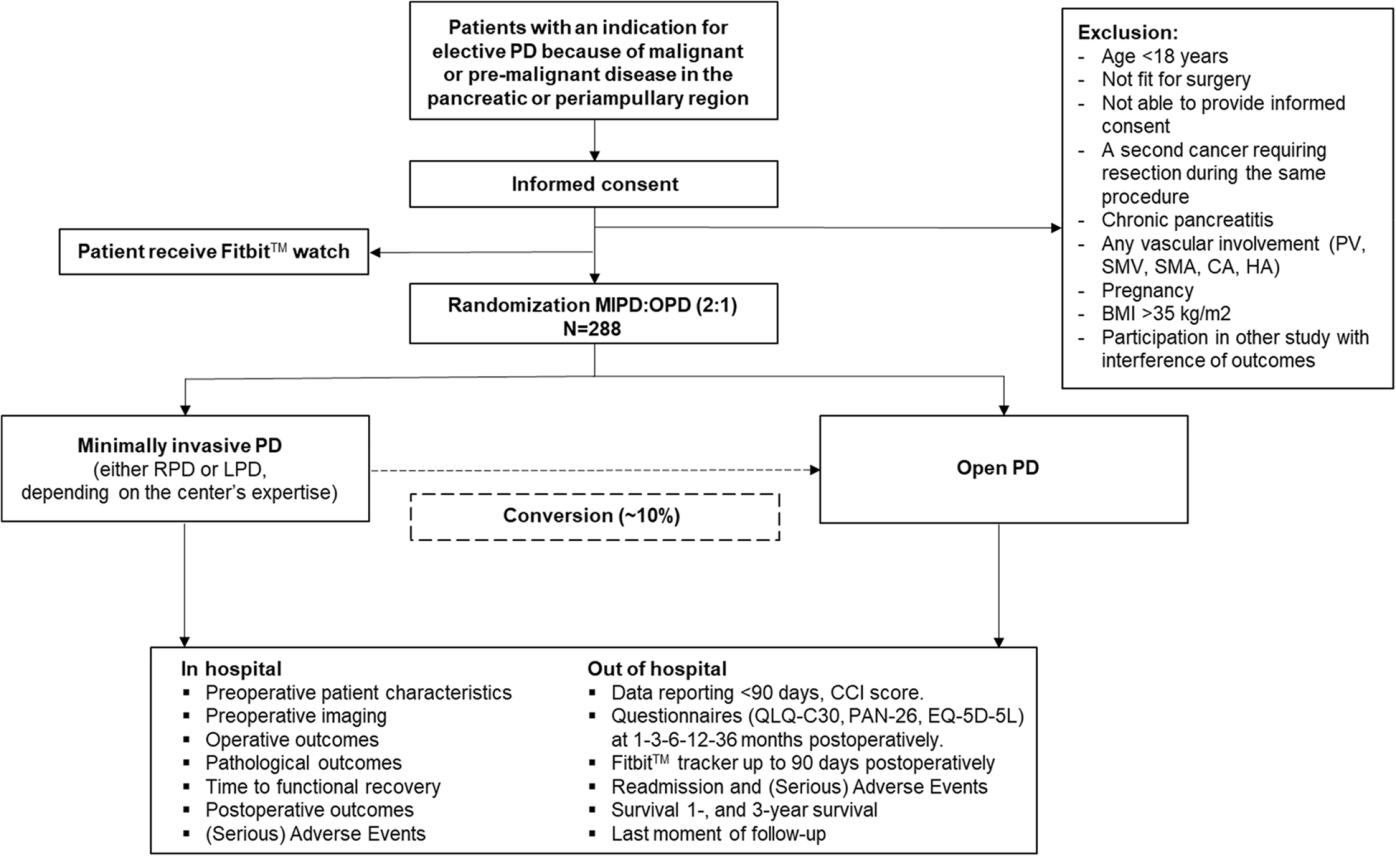
DIPLOMA-2 study flow chart according to SPIRIT

Pancreatoduodenectomy (PD) is a major and complex surgical procedure used to treat various pathologies affecting the pancreatic head, duodenum, ampulla, and distal bile duct and is characterized by a high technical difficulty and challenging postoperative management. Although major improvements in the past decade, such as sub-specialization, centralization, and enhanced recovery protocols, have decreased perioperative mortality, PD remains associated with significant perioperative morbidity which strongly impacts patients’ quality of life as well as healthcare resources. Therefore, much could be gained by optimization of perioperative care and surgical techniques (Fig. 1).

Over the past two decades, many surgical procedures have shifted from the traditional open surgery approach to minimally invasive surgery to reduce surgical trauma, aiming for less postoperative pain, enhanced postoperative recovery, and thereby improved outcomes. Also, minimally invasive pancreatoduodenectomy (MIPD) is increasingly becoming part of clinical practice for selected patients worldwide, as an alternative to open pancreatoduodenectomy (OPD) [1–3]. However, despite promising results from expertise centers, the implementation of minimally invasive techniques for PD is rather slow. This is probably caused by the conflicting outcomes on MIPD as reported by four randomized controlled trials (RCT) on laparoscopic MIPD (L-MIPD) [4–7]. Two single-center and one multicenter RCT showed benefits of L-MIPD in term of less intraoperative blood loss and reduction in length of hospital stay, as compared to OPD, with comparable postoperative morbidity and mortality [4, 5, 7]. However, the preliminary termination of the multicenter LEOPARD-2 trial, comparing L-MIPD and OPD, raised concern on the safety of MIPD due to a higher mortality rate after L-MIPD, diminishing the further implementation of L-MIPD [6]. Since then, the safety of MIPD remains a topic of debate.

Recently, the robotic platform has gained an important place in the surgical field [8]. Especially for pancreatic surgery, robotic surgery has become more popular due to the potential advantages over conventional laparoscopic surgery, including enhanced instrument mobility, 3D visualization, and improved ergonomics [9]. Although promising results on robotic PD (R-MIPD) from high volume expert centers have been reported, no level 1 evidence is available yet, and therefore, the role of R-MIPD is limited to retrospective data from single high-volume centers [10–13].

As MIPD is increasingly being implemented in clinical practice worldwide, a randomized controlled trial (RCT) comparing MIPD to OPD in high-volume centers that have completed the learning curve is needed to demonstrate that MIPD offers improved outcomes that justify the high costs, longer operative times, and learning curve. Furthermore, due to the ongoing uncertainty of the safety and additional benefits of MIPD, many patients affected by pancreatic or peri-ampullary neoplasms might not receive MIPD and do not benefit from potential advantages of an enhanced recovery, such as a reduction of the impact of complications or increased use and completion of adjuvant chemotherapy, compared with OPD.

Therefore, the DIPLOMA-2 trial is designed to primarily investigate the safety of MIPD in terms of morbidity and mortality, and additionally to assess if MIPD is superior to OPD in terms of time to functional recovery, in patients with pancreatic or peri-ampullary neoplasm. Moreover, the DIPLOMA-2 trial aims to compare surgical and oncological outcome, quality of life, and costs after MIPD and OPD. The results of the DIPLOMA-2 trial will answer the present uncertainties regarding safety and guide the further implementation of MIPD worldwide [14].

## Methods

### Design

The DIPLOMA-2 trial is an investigator-initiated, international, randomized controlled patient-blinded non-inferiority trial comparing MIPD versus OPD in patients with a neoplasm located in the pancreatic head or peri-ampullary region. Patients are randomly allocated to MIPD or OPD in a 2:1 ratio, respectively. This unequal randomization provides more statistical power for detecting adverse events in the MIPD group, and secondly will allow for maintenance of a higher annual MIPD volume of the participating surgeons, as MIPD outcomes are strongly associated with hospital volume [15].

Inclusion started after approval of the primary medical ethical review committee board (January 2022). Additionally, local ethical approval was gained for every participating center, before the local initiation of the study. All patients provide a written informed consent before randomization. This protocol was developed according to the SPIRIT guidelines [16].

### Study population

Adult patients with an indication for elective pancreatoduodenectomy because of a proven or suspected neoplasm in the pancreatic head or peri-ampullary region are assessed for eligibility in the DIPLOMA-2 trial. Patient are eligible to participate in case of upfront resectable pre-malignant or malignant disease.

### Inclusion criteria

In order to be eligible to participate in this study, a patient must meet all of the following criteria:

- Age of at least 18 years;
- Indication for elective pancreatoduodenectomy for a proven or suspected* premalignant or malignant neoplasm located in the pancreatic head, distal bile duct, duodenum, or ampulla of Vater;
- Upfront resectable disease (without induction/down-sizing radiotherapy and/or chemotherapy), with pre-operative multiphase CT scan showing no signs of vascular involvement^#^;
- Both MIPD and OPD are considered technically feasible for radical resection, according to the local treatment team;
- The patient is fit to undergo MIPD and OPD according to the operating team;
- Written informed consent given by the patient.

* Pathological proof is not mandatory as it is not common practice in some indications, and the decision for minimally invasive or open surgery after the trial will therefore also depend on the “suspected” diagnosis.

^#^ Neoadjuvant radiotherapy and chemotherapy are allowed in the study, only in case of an upfront resectable tumor. Induction treatment for an initially non-resectable tumor (i.e., locally advanced) is not allowed.

### Exclusion criteria

Patients meeting any of the following criteria will be excluded from participation in this study:

- Second malignancy necessitating resection during the same procedure;
- Chronic pancreatitis as indication or in medical history (according to the M-ANNHEIM criteria [17]);
- Any major vascular tumor involvement (portal vein, superior mesenteric vein, superior mesenteric artery, coeliac artery or hepatic artery) or distant metastases (M1) including involved distant lymph nodes on a CT scan maximum 28 days old;
- Body mass index ≥ 35 kg/m^2^;
- Pregnancy;
- Participation in another study with interference of the primary outcome (CCI®) or time to functional recovery.

### Randomization

Patient recruitment and the collection of written informed consent are performed at the outpatient clinic. Hereafter, all patients will be randomized centrally by the study coordinators using an online computer controlled permuted-block randomization module (Castor EDC, CIWIT B.V., Amsterdam, the Netherlands). Randomization between MIPD and OPD will be performed in a 2:1 ratio, with block sizes varying between 3, 6, and 9 patients. The entire randomization process will be concealed to all involved investigators, except the study coordinator. Randomization will be stratified for the minimally invasive technique (laparoscopic or robotic), the pre-operative risk of post-operative pancreatic fistula (normal versus high risk, based on BMI (< 25 kg/m^2^ and ≥ 25 kg/m^2^) and pancreatic duct diameter (< 3 mm or ≥ 3 mm) on pre-operative imaging), and for the indication (pancreatic ductal adenocarcinoma (PDAC) or other indication). Patients will be coded by a numeric randomization code, and the study coordinator will be the only one with access to it. The source data will be stored digitally and will be kept by the project leader for 15 years after the last patient’s follow-up is completed.

### Surgical technique and postoperative regime

Because of the pragmatic design of the DIPLOMA-2 trial, no specific standards for MIPD and OPD are provided. All procedure details will be recorded within an online case record form immediately after surgery. There are no restrictions regarding postoperative care, blood tests, drain management, the use of medication, or other kinds of co-intervention. However, the participating centers should provide the same postoperative care for both study arms, based on enhanced recovery principles, which include early mobilization and expanding oral intake as desired by the patient. The treating team will be asked to specify the use of this kind of additional (surgical) proceedings and medication in the online case record forms.

### Conversion from MIPD to OPD

Any incision used for other reasons than trocar placement and specimen extraction is defined as a conversion. Patients allocated to MIPD but converted to OPD will still be analyzed in the MIPD group, according to the intention-to-treat principle. Reasons for conversion will be registered and categorized as urgent or non-urgent conversions [18].

### Blinding

Within the DIPLOMA-2 trial, it was not deemed possible to blind the assessors of the primary outcome, due to logistical reasons. Therefore, an independent adjudication committee, blinded for treatment allocation, will assess the primary outcome of all included patients. Furthermore, regarding the most important secondary outcome, time to functional recovery, patients will be blinded for treatment allocation pre-operatively, until 5 days after surgery. Directly after skin closure, while the patient is still under general anesthesia, the patients will receive a firmly taped, large 40 × 40 cm abdominal dressing to cover their incision(s), and therefore, their treatment allocation will remain blinded (minimally invasive or open). This abdominal dressing will be removed at postoperative day 5, or earlier when all criteria for functional recovery are met, or for medical reasons, such as suspicion of wound infection. If earlier inspection is required, attempts are made to maintain patient blinding. This blinding has proven to be successful in previous multicenter RCTs [19–21]. The success of blinding will be assessed using the blinding index as proposed by Bang et al. [22] Patients will be asked on day 2 and before removal, about the alleged treatment allocation, based on five categories: (1) strongly believe it was MIPD, (2) somewhat believe it was MIPD, (3) do not know, (4) somewhat believe it was OPD, (5) strongly believe it was OPD. Patient blinding will not be performed in patients who are intra-operatively diagnosed with irresectable disease, such as metastases. Since patient blinding only influences one of the secondary outcomes, time to functional recovery, and not the primary outcome, this is considered of minor influence. Sensitivity analysis, excluding patients with perioperative diagnosed metastasized disease, will be performed for analysis of time to functional recovery.

### Primary outcome

The primary outcome is the Comprehensive Complication Index (CCI®), measured up to 90 days post-operatively. The CCI® score is developed to reflect the postoperative morbidity based on the Clavien-Dindo classification of postoperative complications and is validated for pancreatic surgery [23–25]. The CCI® score ranges from 0 (no complication of any kind during the postoperative period) and 100 (death of patient). The advantage of the CCI® over the Clavien-Dindo classification is the ability to accumulatively assess the postoperative morbidity considering all complications instead of only scoring the “most severe” complication. Repeat Clavien-Dindo 1 complications will be counted only once (i.e., not be scored cumulatively) in the CCI.

### Secondary outcomes

The most important secondary outcome is postoperative time to functional recovery. Functional recovery, as defined by a previous RCT [19, 20], is reached when all of the following criteria are met: (I) adequate pain control with oral analgesia only; (II) restoration of mobility to an independent level (or to preoperative level if previously impaired); (III) ability to maintain sufficient caloric intake (minimum of 50% required calories); (IV) absence of intravenous fluid administration; (V) no signs of active infection (no fever, decreasing C-reactive protein below 150 mg/L).

Post-operative activity will be measured using a Fitbit™ Inspire 2, by patients on their wrists (1–2 weeks) from prior to surgery to 90 days after surgery [26–29]. A comprehensive overview of the outcomes of the postoperative activity tracking will be described as a separate publication.

Other secondary outcomes of this trial include intra-operative parameters (type of surgery (laparoscopic or robot-assisted), conversion (urgent or non-urgent), method of anastomosis, vessel resection, operative time, blood loss, and blood transfusion), postoperative outcomes (up to 90 days after surgery; major complications (defined as a Clavien-Dindo grade III or higher) and mortality (and whether related to surgery), postoperative pancreatic fistula [30], post-pancreatectomy hemorrhage [31], delayed gastric emptying [32], chyle leak [33], surgical site infection [34], postoperative intervention (surgical, radiologic or endoscopic), intensive care unit admission, (multi) organ failure, length of hospital stay, readmission, (time to) start of adjuvant therapy) and pathological outcomes (pathological diagnosis, tumor size, histology and tumor grading [35], distance from the tumor to all margins, number of retrieved lymph nodes, number of positive lymph nodes, lymphovascular and perineural tumor invasion, and venous and arterial tumor involvement). For the economical evaluation, costs (intra-operative and postoperative costs (up to 90 days)) and quality of life (using the validated EQ-5D-5L, QLQ-C30, and PAN-26 questionnaires with additional questions regarding scar complications and body image (up to 3 years)) are compared [36, 37].

### Data collection and patient follow-up

Baseline characteristics (age, sex, performance status (Karnofsky score [38]), ASA physical status, body mass index, previous abdominal surgery, preoperative diabetes mellitus, preoperative imaging conclusion including tumor size and involvement of other organs and vessels, neo-adjuvant treatment, serum levels of Hba1C, CA 19.9 and CEA, and baseline quality of life questionnaires’ scores will be recorded before intervention. All required clinical data will be collected after randomization (i.e., from hospitalization up to 36 months postoperatively) using standardized online case report forms by the local treating physicians and will be crosschecked with source data by the study coordinators at 3 and 36 months after the last patient is enrolled. To assess postoperative daily activity (up to 90 days), this study uses the Fitbit™ Inspire 2 to record step counts (as a measure of physical activity), and heart rate, combined as “active minutes” per day. For quality-of-life measurements, the validated questionnaires will be sent (electronically) to participating patients at baseline and at 1, 3, 6, 12, and 36 months after surgery. Patients will also receive an additional questionnaire at 6 months focusing on possible complications with the scar and body image. Patients will be followed at the outpatient clinic according to local protocols (including abdominal CT scan and serum levels of CA 19.9 and CEA tumor markers, if indicated). In each center, a monitor visit is performed 90 days after the last included patient has undergone surgery to cross-check primary and secondary outcome data.

### Quality and safety

A minimum annual center volume of at least 30 MIPD and 30 OPDs was set as requirement for participating in the DIPLOMA-2 trial. By using this volume requirement, we aim for centers to maintain an annual volume of at least 20 MIPD during the trial, despite randomization [14]. Centers will participate for either L-MIPD or R-MIPD, depending on their main expertise. Surgeons are allowed to participate in DIPLOMA-2 if they have a personal experience of at least 60 MIPDs and 60 OPDs in the past 10 years.

All MIPD surgeons will be required to have participated in an endorsed MIPD training program or otherwise are asked to send a recorded and anonymized video of a personal MIPD procedure performed, before the start of the trial, which will be evaluated by the study team. In addition, the MIPD procedures within the DIPLOMA-2 trial will be recorded and stored, to be available upon request of the study team.

All adverse events will be recorded up to 90 days postoperatively. Serious adverse events will be reported through a web portal (www.toetsingonline.nl) to the Dutch central committee on research involving human subjects (in Dutch: Centrale Commissie Mensgebonden Onderzoek (CCMO)) and the institutional review board (Medical Ethics Committee of Amsterdam UMC). The following serious adverse events must be reported to the study coordinator within 24 h: unplanned intensive care unit admission, any surgical intervention, readmission, and death (regardless of cause). The remaining adverse events are recorded in a yearly overview list. An independent data safety monitoring board (DSMB) is appointed to evaluate the study safety parameters. When each 50th included patient has completed 90 days of follow-up, the DSMB will meet (online) in order to assess the safety parameters. The DSMB exists of one independent statistician, one independent methodologist, one independent medical oncologist, and three independent surgeons. One of the DSMB members is appointed as chairman and a second member as secretary. The minutes of these meetings will be sent to the institutional review board of the study by the study coordinator and the trial steering committee. The DSMB will not be blinded and will be fully informed on all SAEs. The DSMB can request a full report of specific study outcomes whenever required. The study coordinator and principal investigator will only be present during the start (open discussion) of the DSMB meeting to provide the overall data and provide background information.

### Ethics

The DIPLOMA-2 trial will be conducted according to the principles of the Declaration of Helsinki (64^th^ version, October 2013) and in accordance with the local laws and regulations, such as in the Netherlands the Medical Research Involving Human Subjects Act. The local principal investigators are responsible to adhere to local laws and regulations. The independent ethics review board of the Amsterdam UMC (Amsterdam, the Netherlands; NL77750.018.21) and Provincial Ethic Committee Brescia (Brescia, Italy; NP4916) have approved the study protocol. Furthermore, approval from all local ethics committees of participating centers is obtained before participation in the study. The trial is registered in the ISRCTN registry: ISRCTN27483786.

### Statistical aspects

#### Sample size calculation

The DIPLOMA-2 trial is designed as a non-inferiority trial, hypothesizing that the mean CCI® score of MIPD is non-inferior to OPD. Calculations are based on results of the pan-European E-MIPS retrospective cohort study and on individual patient-data collected from the previous conducted RCTs on MIPD versus OPD. The sample size is calculated using PASS 2022 software to achieve 80% power (1-β) in a per-protocol analysis with the following assumptions: 2.5% one-sided significance level (α), expected difference of the mean CCI® score in the MIPD and OPD group of 0 points, and a non-inferiority margin of 7.5 points, standard deviation of 20 points in both groups, including 5% metastasized disease, a 10% conversion rate and a 3% lost-to-follow-up rate after randomization leads to a total number of patients to be randomized of 288 (192 in the MIPD study arm and 96 in the OPD study arm). For time to functional recovery, we assume MIPD will result in a reduction of 2 days (7 days in the MIPD group versus 9 days in the OPD group. Time to functional recovery will be measured in days and will be tested for superiority using the Wilcoxon (Gehan-Breslow) test or the log-rank test (based on non-normal distribution). With a one-sided testing, assuming 5% metastatic disease, 10% conversion rate, and 3% loss to follow-up, a sample of 247 patients (159 in the MIPD group and 88 in the OPD group) in a per protocol analysis achieves 80% power to detect a 28% reduction of time to functional recovery (i.e., 6.5 days in the MIPD group; hazard ratio = 0.72). The estimated median time to functional recovery of 9 days in the OPD group is based on results of the LEOPARD-2 trial and corresponds to a median TTFR reduction of 2.5 days.

### Statistical analysis

Primary and secondary endpoints will be cross checked with data from primary sources, and a blinded adjudication committee will check them against the used definitions. Primary and secondary endpoint data of all randomized patients will be analyzed based on three analysis sets. The modified intention-to-treat (mITT) set comprises all patients who underwent surgery in the group to which they were randomized (converted patients remain in the MIPD group, excluding patients who were excluded prior to surgery) and serves as the primary analysis set. The per-protocol (PP) set consists of all patients treated per protocol without major protocol violation and without conversions. In addition, the as-treated set will be analyzed, considering the patients in the group in which they were finally treated (i.e., converted patients in the OPD group). There is no evidence that the converted patients will have a higher postoperative CCI® than those patients randomized to and remaining in the OPD group. Therefore, the as-treated set is an important complement to the ITT and PP sets in this trial.

The primary outcome measure “CCI®” will be expressed as means (standard deviations) or medians (interquartile ranges) and will be tested for non-inferiority using independent samples *t*-test or Mann–Whitney *U* test, as appropriate dependent of the distribution. The most important secondary outcome measure “time to functional recovery” will be measured in days and will be tested for superiority using the Wilcoxon (Gehan-Breslow) test or log-rank test, depending on the distribution. Secondary analysis will include correction for censored patients (death before time to functional recovery obtained). For the primary study outcome, the lower limit of the two-sided 95% confidence interval of the difference in proportions will be reported and compared with the non-inferiority margin. Additionally, the individual functional recovery criteria will be analyzed separately. Major complications, expressed in proportions, will be tested for non-inferiority using chi-square or Fisher’s exact test as appropriate. The distribution of variables will be determined using several plots (boxplot, Q-Q plot and histogram) and the Kolmogorov–Smirnov and Shapiro–Wilk tests. For comparison of normally distributed continuous variables, the independent samples *t*-test will be used, and values will be expressed as means (standard deviations). Continuous non-normally distributed variables will be compared using the Mann–Whitney *U* test, and values will be expressed as medians (interquartile ranges). Categorical variables will be compared by chi-square or Fisher’s exact test as appropriate, and values will be expressed as proportions. A two-tailed *p*-value < 0.05 will be considered statistically significant. Time to event endpoints, such as survival, will be calculated using Kaplan–Meier estimations. A Cox regression analysis will be performed to investigate predictors of postoperative survival. All parameters with a *p*-value < 0.1 in a univariable analysis will be included in the multivariable Cox regression analysis. Additionally, multivariable analyses will be performed to determine predictors for primary and secondary study outcomes, for example the occurrence of major complications, postoperative pancreatic fistula, and R0 resection. Furthermore, predictors for receiving adjuvant chemotherapy or readmission will be assessed.

For subjects who are lost to follow-up, a sensitivity analysis will be performed to determine best case/worst case scenarios.

The following pre-specified subgroup and sensitivity-analyses will be performed:

- Comparing MIPD and OPD in patients with major complications (Clavien-Dindo grade ≥ III) and without major complications (no complication or Clavien-Dindo I-II) separately
- Comparing centers for low and high annual MIPD volume during the trial separately (< 20 MIPD/year versus ≥ 20 MIPDs/year)
- Comparing MIPD and OPD in patients with pre-malignant indications and PDAC separately.
- Comparing incidence of POPF in MIPD and OPD for the ISGPS subgroups of POPF risk and the A-B-C variant [39, 40].
  o A—not soft (hard) texture and MPD > 3 mm
  o B—not-soft (hard texture and MPD ≤ 3 mm
  o C—soft texture and MPD > 3 mm
  o D—soft texture and MPD ≤ 3 mm
- Comparing R-MIPD and OPD for the primary outcome CCI®, time to functional recovery, and other outcomes
- Comparing L-MIPD and OPD for the primary outcome CCI®, time to functional recovery, and other outcomes
- Comparing R-MIPD and L-MIPD for the primary outcome CCI and time to functional recovery 

A detailed statistical analysis plan will be drafted prior to database lock of the short-term outcomes (up to 90 days postoperative). Despite all prior preventive measures taken, a complex international trial may evoke unforeseen situations after database lock that threaten data integrity and can only be resolved by unlocking the database prior to the final analysis. For purpose of transparency and reproducibility, the statistical analysis plan will therefore also describe the procedure to be followed when such situations arise.

### Dissemination policy

The results of this trial will be submitted to a peer-reviewed medical journal regardless of the study outcome. Authorship will be based on international guidelines. Those involved with the study who do not fulfil these criteria will be listed as “collaborator.”

## Discussion

The DIPLOMA-2 trial is an international randomized controlled, patient-blinded trial assessing the non-inferiority of MIPD compared to OPD regarding overall complications in patients with any pancreatic and peri-ampullary neoplasm. The study was carefully designed and initiated by the European Consortium on Minimally Invasive Pancreatic Surgery (E-MIPS), to ensure the generating of high-quality data that can be used to guide clinical practice and improve patient outcomes. The DIPLOMA-2 trial will represent a significant advancement in the field of pancreatic surgery, as it is the first international multicenter patient-blinded randomized controlled trial comparing MIPD with OPD for premalignant and malignant disease.

To date, four randomized studies on L-MIPD versus OPD have been published, of which two single center studies, and two multicenter studies [4–7]. Three studies reported that LPD was safe and feasible, with lower blood loss, shorter hospital stay, and faster recovery with similar mortality and mortality, as compared to OPD. However, the LEOPARD-2 trial was terminated early because of a trend towards higher mortality rate in the L-MIPD group (5 (10%) vs 1 (2%), *P* = 0.20) which raised questions regarding the safety of this approach. This led to a reduction in the use of L-MIPD worldwide and ever since the question of the safety of MIPD versus OPD remained a topic of debate [41]. For this reason, the E-MIPS consortium collectively decided that in order to safely and validly expand MIPD worldwide, a new large international multicenter trial in experienced high-volume centers with strict quality standards was needed, with the necessary modifications to the design.

For this reason, the primary outcome of the DIPLOMA-2 trial is overall complications, measured using the CCI®. This choice was based on the various (online) meetings with all principal investigators involved, where there was consensus on safety as the most clinically relevant endpoint for patients undergoing MIPD. By focusing on cumulative complications, the trial aims to capture a comprehensive picture of the safety and efficacy of MIPD compared to OPD. Because the expected benefits of MIS lie in the reduction of surgical impact, and therefore enhanced postoperative recovery, the trial is also powered to assess superiority of time to functional recovery, as a most important second outcome measure.

In order to ensure the quality and safety within the DIPLOMA-2 trial, several strict requirements have been established for participating centers and surgeons. First, a minimum annual center volume of 30 MIPD and 30 OPD was set as a requirement for participation in the trial. This volume requirement was set to ensure that participating centers maintain an annual volume of at least 20 MIPD during the trial, despite of the volume reduction caused by randomization, as advised by the Miami guidelines [14]. This is important because MIPD outcomes have been shown to be strongly associated with hospital volume. In addition to the center requirements, participating surgeons were required to have a minimum of 60 MIPDs and 60 OPDs of experience in order to participate in the trial. All MIPD surgeons are also required to have participated in an endorsed MIPD training program or, alternatively, were asked to send a recorded and anonymized video of a personal MIPD procedure that would be evaluated by the study team. These measures were taken to ensure that the participating surgeons were highly skilled and experienced in conducting MIPD and OPD procedures. By allowing only experienced centers and surgeons to participate, we believe we can better investigate the true (potential) effect of the MIPD versus OPD on complications, lowering the confounding effect of the learning curve. All MIPD procedures will be recorded and stored, to be available upon request of the study team. These recordings will allow the study team to review and evaluate the procedures to ensure that they were conducted according to the trial protocol and that any adverse events will be properly documented.

When constructing RCTs, blinding—the practice of concealing the allocated intervention—is known to be an important aspect in preventing the introduction of bias [42]. Patients included in DIPLOMA-2 will therefore be blinded for the surgical approach, from randomization up to 5 days after surgery. This method of blinding is successfully used in previous trials on minimally invasive versus open surgical procedures and is applied to minimize any potential bias in the stimulus of postoperative activity of patients between the two surgical approaches and to increase the validity of the results regarding functional recovery [6, 43, 44]. Additionally, the blinding of participants before the surgery may also aid participant retention and compliance.

A frequent criticism on surgical randomized controlled trials, particularly when comparing a novel intervention to a conventional intervention, is that the comparison may be inherently “unfair” due to a difference in expertise of the participating surgeons. An imbalance in the surgical learning curve between MIPD and OPD could lead to incorrect conclusions, especially when focusing on the safety aspect of the two procedures. In the DIPLOMA-2 design, we tried to control for the impact of this learning curve by the entry criteria for centers and surgeons, and using the 2:1 randomization, which allows for maintenance of a higher annual MIPD volume.

## Conclusion

In conclusion, the international randomized controlled, patient-blinded DIPLOMA-2 trial is designed to investigate the non-inferiority of MIPD versus OPD for overall complications (primary endpoint). Secondly, the trial is powered to analyze the superiority of MIPD versus OPD regarding time to functional recovery (secondary endpoint). If successful, the DIPLOMA-2 trial may establish MIPD as the new standard of care for patients undergoing pancreatoduodenectomy in this selected patient population in selected high volume centers following the Miami guidelines. When demonstrating the safety and efficacy of MIPD, the trial may help to expand and standardize the use of MIPD, ultimately leading to better patient outcomes.

### Trial status

**Fig. 2:**
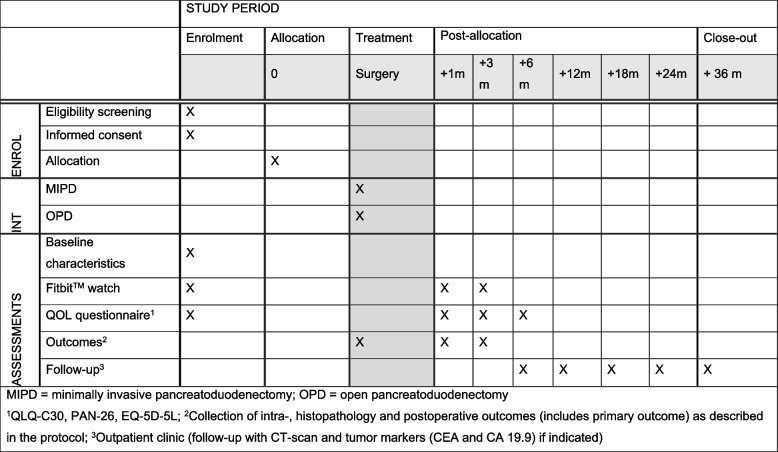
Schedule of enrolment, interventions, and assessments according to SPIRIT

Confirmation of funding of the trial by Intuitive Surgical Sarl. was received on December 17, 2020. Ethical approval of the primary review committee (Amsterdam UMC) was received on December 03, 2021. The DIPLOMA-2 trial was published in the ISRCTN registry on January 11, 2021 (ISRCTN 27483786). The first patient underwent surgery at January 03, 2021. At the time of submitting this protocol for publication (June 14, 2023), all 14 centers were actively recruiting patients for the trial, and 269 out of 288 (93%) were randomized, of which 257/288 (89%) underwent surgery, which means that inclusion is ahead of schedule (Fig. 2).

## Data Availability

Not applicable.

## Abbreviations

MIPD: Minimally invasive pancreatoduodenectomy
OPD: Open pancreatoduodenectomy
CCI: Comprehensive complication index
R0: Microscopically radical resection
PDAC: Pancreatic ductal adenocarcinoma
CCMO: Centrale Commissie Mensgebonden Onderzoek
DSMB: Data Safety Monitoring Board
SAE: Serious adverse event
BMI: Body mass index
PV: Portal vein
SMV: Superior mesenteric vein
SMA: Superior mesenteric artery
CA: Coeliac artery
HA: Hepatic artery
